# Modulation of the CCR6-CCL20 Axis: A Potential Therapeutic Target in Inflammation and Cancer

**DOI:** 10.3390/medicina54050088

**Published:** 2018-11-16

**Authors:** Ranmali Ranasinghe, Rajaraman Eri

**Affiliations:** School of Health Sciences, College of Health and Medicine, University of Tasmania, Launceston, Tasmania 7248, Australia; ranmaliranasinghe301@gmail.com

**Keywords:** CCR6, CCL20, inhibitors, T_H_17, T_reg_, inflammatory diseases, cancer

## Abstract

Prototypical functions of the chemokine receptor CCR6 include immune regulation by maneuvering cell chemotaxis and selective delimiting of the pro-inflammatory T_H_17 and regulatory T_reg_ subsets during chronic or acute systemic inflammation. Inhibition of CCR6 is proposed to attenuate disease symptoms and promote recuperation of multiple inflammatory and autoimmune disorders. Prescription medicines with pharmacodynamics involving the inhibition of the chemokine axis CCR6–CCL20 are very limited. The development of such therapeutics is still at an early experimental stage and has mostly involved the utilization of pre-clinical models and neutralizing mono or polyclonal antibodies against either partner (CCR6 or CCL20). Other methods include the constitutive use of small molecules as peptide inhibitors or small interfering ribonucleic acid (siRNA) to interfere with transcription at the nuclear level. In our review, we aim to introduce the wide array of potential CCR6–CCL20 inhibitors with an emphasis on attendant immune-modulator capacity that have been tested in the research field to date and are immensely promising compounds as forerunners of future curatives. Sixteen different tractable inhibitors of the CCR6–CCL20 duo have been identified as possessing high medicinal potential by drug developers worldwide to treat autoimmune and inflammatory diseases as shown in Figure 1. A multitude of antibody preparations are already available in the current pharmaceutical market as patented treatments for diseases in which the CCR6–CCL20 axis is operative, yet they must be used only as supplements with existing routinely prescribed medication as they collectively produce adverse side effects. Novel inhibitors are needed to evaluate this invaluable therapeutic target which holds much promise in the research and development of complaisant remedies for inflammatory diseases.

## 1. Introduction

Chemokines are chemoattractant cytokines that consist of small molecular proteins that are integral to the immune system. The quintessential roles of chemokines include the balance of immune system integrity during steady state homeostasis and the maintenance of host defense during inflammatory onslaughts. Chemokines bind to their cognate receptors to discharge their immune modulatory functions in a concerted fashion aided by the local cytokine milieu—adhesive and co-stimulatory molecules within the tissue microenvironment. Chemokines may be non-promiscuous, i.e., bind with a sole ligand, or redundant, where multiple receptors bind to one ligand or vice versa. To date, around fifty chemokine ligands and twenty receptors with molecular weights of 8–14 kDa have been identified. Chemokines and their receptors are essential for trafficking leukocytes during immune responses and orchestrating immune cell chemotaxis to inflammatory sites. They are also involved in a host of other functions, such as embryonic development, angiogenesis, wound healing, T helper subset development, B cell maturation, and differentiation. Chemokines and receptors are present extracellularly on T and B lymphocytes, dendritic cells, macrophages, monocytes, neutrophils, eosinophils, basophils, innate lymphoid cells, neurons, epithelial, and endothelial cells. Chemokine-mediated immune cell migration also contributes to multiple autoimmune and chronic inflammatory diseases as well as cancer metastasis and infection by human immunodeficiency virus (HIV) [1,2,3,4,5,6,7].

Among the chemokine repertoire, the CC chemokine receptor–ligand pair, CC chemokine receptor 6 (CCR6) and CC chemokine ligand 20 (CCL20) is becoming recognized for its invaluable therapeutic potential in immunological research. CCR6 is a 7-transmembrane domain G protein-coupled receptor, and CCL20 is known by several names, such as macrophage inflammatory protein MIP-3α, Exodus-1, and liver and activation regulated chemokine (LARC). CCL20 is expressed only by T helper lymphocyte 17 (T_H_17) cells and not by regulatory T cells or other T helper subsets. The importance of the CCR6–CCL20 axis was emphasized by the discovery of the CCR6-expressing CD4^+^ T helper cell sub populations, T_H_17, and regulatory T_reg_ cells, which form a platform that stringently regulates immune tolerance in healthy individuals. Disruption of this delicate alliance will tip the balance in favor of either the pro-inflammatory signature cell population, T_H_17, or its immune regulatory partner, the regulatory T_reg_ cells, which are avidly recruited to the sites of infection or injury [8,9,10,11].

**Figure 1:**
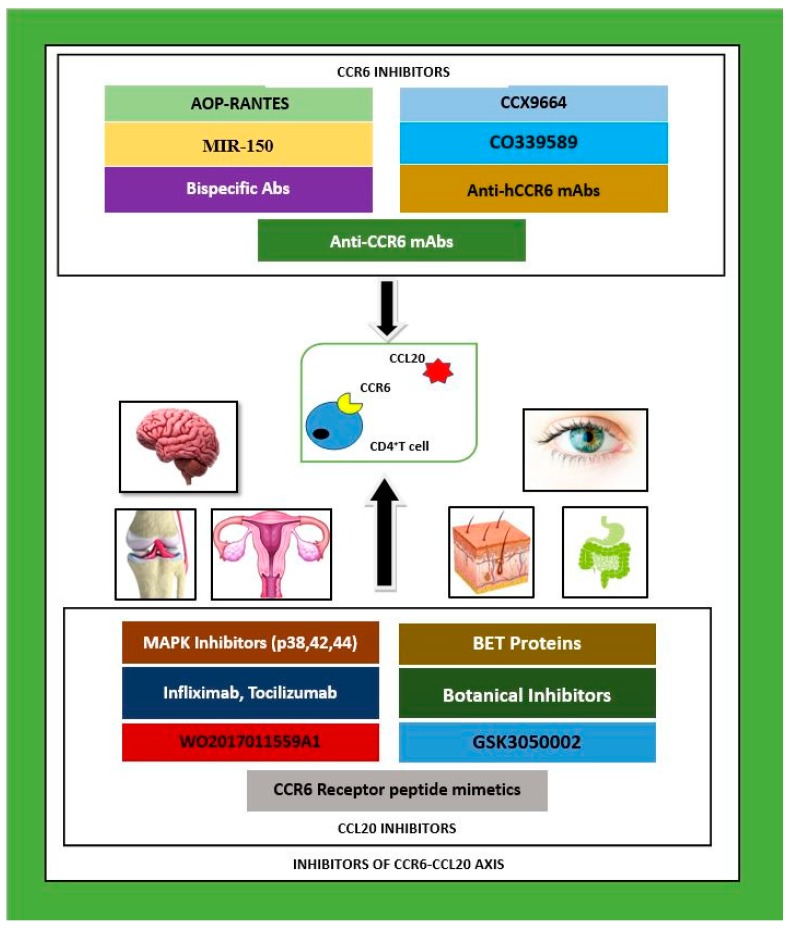
CCR6–CCL20 axis inhibitors investigated in pre-clinical and clinical studies to date as possible therapeutics for autoimmune and inflammatory diseases in multiple organ systems: nervous, skeletal, integumentary and gastrointestinal systems. CCR6 inhibitors are given above as a cluster, and CCL20 inhibitors are given below as a cluster. Legend: MAPK—mitogen activated protein kinase, p—protein, CCL20—CC chemokine ligand 20, mAbs—monoclonal antibodies, CCR6—CC chemokine receptor 6, hCCR6—humanised CCR6, Abs—antibodies, IL—interleukin, BET—bromodomain extraterminal proteins, R—receptor, MIR-150—micro ribonucleic acid-150, AOP-RANTES—amino oxypentane regulated on activation, normal T cell expressed and secreted.

CCR6 and CCL20 have exhibited characteristics in tune with both immune homeostasis and immune activation. It is already established that the immunological impact of this chemokine receptor–ligand partnership has far reaching consequences in health and disease that affect multiple organs of the human body. A plethora of research studies have demonstrated that the CCR6 and CCL20 axis directly influences the nervous, respiratory, gastrointestinal, excretory, skeletal, and reproductive systems via pleiotropic immune mechanisms, manifesting as diseases with high mortality rates. Given the exclusive roles that CCR6 and CCL20 play in clinical pathophysiology, this chemokine pair is considered as a potential therapeutic target, whereby the blockade or inhibition of either partner as also shown in Figure 1 and supported by Table 1 is expected to produce successful pharmacotherapy as a treatment for its related diseases [12,13,14,15,16].

## 2. Inhibitors of CCR6–CCL20

CCR6–CCL20 axis inhibitors include (i) antibodies that bind CCL20; (ii) antibodies that bind CCR6; (iii) small molecule inhibitors of CCL20; (iv) small molecule inhibitors of CCR6; (v) small interfering RNA (siRNA) that hybridizes to a nucleic acid encoding CCL20 and (vi) siRNA that hybridizes to a nucleic acid encoding CCR6 [17]. Most of the studies undertaken up until now depended heavily on the utilization of neutralizing monoclonal or polyclonal antibodies.

Current prescription medicines consist of disease-modifying drugs which ameliorate symptoms and pathology through non-specific suppression of inflammatory pathways. The blockade of inflammatory cytokines and products that up-regulate pro-inflammatory chemokines is known to further induce inflammation. The biological agents Infliximab, Tocilizumab, and Etanercept, have been used to block CCL20, which is otherwise known to markedly increase inflammation in autoimmune and inflammatory disorders. Infliximab is an anti-tumor necrosis factor-alpha (TNF-α) monoclonal antibody that is used to neutralize the inflammatory cytokine TNF-α; Etanercept has an anti-TNF-α modified as a fusion protein; and Tocilizumab is an antibody produced against interleukin-6 receptor (IL-6R). All three of these agents have demonstrated a significantly reduced CCL20 concentration below serum baseline values, and they are combined as supplements as a routine pharmacotherapy. Infliximab depresses TNF-induced CCL20 upregulation via the nuclear factor kappa B (NF-kB) pathway in synoviocytes which causes rheumatoid arthritis. Blockade of IL-6 modulates the CCR6–CCL20 axis by preventing the differentiation of T_H_17 cells from CD4^+^ T cell populations [18].

Early studies on the specific blocking of either CCR6 or CCL20 included the transfer of CD4^+^ T cells from Sakaguchi mice into severe combined immunodeficiency (SCID) mice and the administration of a monoclonal antibody (mAb) against CCR6 in a rheumatoid arthritis model. The mAb administered mice exhibited lowered migration of T_H_17 cells to the joints with a recognizably reduced disease severity. Other research has mostly pivoted around CCR6^−/−^ mice, pointing in the direction of a disrupted CCR6–CCL20 axis which affects immune cell chemotaxis and a discernible change in immune homeostasis. In the guts of inflammatory bowel disease (IBD) models, CCR6 deficiency indicated pronounced changes in the overall histology at the Peyer’s patches [18].

Chemokines bind with their G protein coupled receptors (GPCR) via an extensive protein-protein interface that includes domains at the extracellular surface and a deep pocket within the transmembrane domain (known as the orthosteric site) at the N terminus. Native chemokines, which are constitutive GPCR agonists, can elicit intracellular responses, including directional cell migration through the activation of heterotrimeric G proteins and the β-arrestin-mediated signal transduction pathway. CCR6 is a seven-transmembrane domain GPCR which binds with its sole ligand, CCL20, and innervates tactical lymphocyte migration to sites of inflammation in the same way. The therapeutic strategies explored so far have employed the inhibition of chemokine signaling by small molecule or peptide antagonists, which are typically engineered to block GPCR signaling by binding at the orthosteric site and preventing activation by the corresponding chemokine ligand [19].

Partial or biased agonists of chemokine receptors which exhibit a loss of efficacy in certain types of signaling pathway can also be engaged as potent inhibitors, for example, the amino oxypentane-regulated activation, normal T cell expressed and secreted (AOP-RANTES) pathway, a modified form of regulated activation, and the normal T cell expressed and secreted/CC chemokine ligand 5 (RANTES/CCL5) pathway were reported by Getschman and colleagues. AOP-RANTES induces CCL5-mediated calcium signaling but lacks pro-migratory signaling and has altered receptor recycling properties. The increased efficacy of engineered chemokines with partial agonist activity is displayed by the greater anti-HIV potency of AOP-RANTES compared to native RANTES, and these chemokines act as alternatives to small molecule GPCR antagonists [19]. Changing the chemokine’s oligomeric state can significantly alter its signal transduction properties. In vivo chemokine function essentially depends on binding to glycosaminoglycans present in the extracellular matrix, and the self-association of chemokines is known to relatively enhance this binding activity. Although full GPCR activity happens in the monomeric state, two conserved types of dimerization exist in the CC and CXC chemokine subfamilies, and as proof of dimeric functionality, two CCL20 variants designed to form dimers acted as partial agonists for the chemokine receptor CCR6. In a murine model of interleukin-23 (IL-23) dependent psoriasiform dermatitis, two novel dimeric CCL20 variants induced (i) intracellular calcium release, (ii) minimized chemotactic activity, (iii) inhibited CCR6-mediated T cell migration, (iv) up-regulation of interleukin-17 (IL-17) and interleukin-22 (IL-22), and (v) the prevention of psoriatic inflammation, thus highlighting the role of CCR6 as a controllable therapeutic target [19].

Chemokine receptors have been shown to employ several different pathways in inflammatory and autoimmune disorders and hence, it is advisable to simultaneously target more than one receptor–ligand pair to evaluate their efficacy in disease suppression. Fully humanized, immunoglobulin-G (IgG)-like bispecific antibodies (BsAb) have been developed against CXCR3 and CCR6 and their validity has been tested as a promising therapeutic target which (i) regulates cell migration of the T_H_17 subset of CD4^+^ T lymphocytes; (ii) effectively blocks cell chemotaxis; and (iii) induces specific antibody-dependent cell-mediated cytotoxicity in vitro. Dual targeting of chemokine receptors with a fully humanized BsAb in this instance thus provides a potent interventional approach for the treatment of inflammatory and autoimmune diseases. Depletion of various pro-inflammatory subsets of pathological lymphocytes is another therapeutic approach, but broad targeting of immune cell migration could turn out to be immune compromising. Thus, a combination of blocking agents may be required to prevent the recruitment of mixed leukocyte subsets to inflamed tissue to reap the maximal therapeutic benefits [20].

The use of anti-inflammatory botanical inhibitors as antagonists of CCR6 and CCL20 has been studied. Epigallocatechin gallate (EGCG), a polyphenol compound found in green tea was reported to inhibit CCL20-mediated cell chemotaxis by a significant amount (20%) in a study involving recombinant and chemically synthesized rhesus macaque chemokines that were developed for studying interactions between CCR6 and CCL20. An inhibition rate of CCL20-driven chemotaxis of 100% was observed with gallotannin, a phenolic metabolite (tannin) that occurs naturally in foods, such as chick and cowpeas, nuts, mangoes and rhubarb, and is associated with the scavenging of free radicals and interference of both herpes virus and HIV. The same study also reported that the CCR6 receptor peptide mimetic extracellular loop-2 (ECL-2) inhibits CCL20 induced migration. Extracellular loops of chemokine receptors called receptor peptide mimetics or ECL-X (where X refers to the number of sequential extracellular loops) can bind directly to chemokines and inhibit chemokine-induced migration [21].

## 3. CCR6–CCL20 Inhibition in Multiple Organ Systems

### 3.1. Nervous System

CCR6 plays a specific role in the neuroinflammatory response in experimental autoimmune encephalitis (EAE). This was validated by the delayed onset and reduction of disease observed in CCR6^−/−^ mice compared to wild type (WT) mice. In an experimental model of EAE, CCR6 was implicated not so much as having an effector function, but as having a role in the enhanced priming of T helper cells. The study demonstrated impaired development of EAE in *Ccr6* deficient mice and mice treated with a neutralizing anti-CCR6 antibody (Ab) or novel CCR6 antagonist bearing synthetic truncated CCL20 peptides. Three functional outcomes were determined by this research, which are summarized as (i) CCR6 is critical for the priming phase of EAE; (ii) the recruitment of immature dendritic cells (DCs) to tissue is CCR6 dependent and acts as a limiting factor for T cell priming; and (iii) CCR6 regulates lymphocyte egress from peripheral lymph nodes during active immune stimulation [22].

Currently, no effective mAb inhibitors against CCR6 exist for use in mouse models of inflammation, but this has been circumvented by the use of transgenic mice (Tg/m) expressing human CCR6 (hCCR6) under the control of their native promoter (hCCR6-Tg/mCCR6^−/−^). Anti hCCR6 mAb was recognizably effective in reducing disease severity in EAE by remarkably attenuating the clinical symptoms of myelin oligodendrocyte glycoprotein (MOG) induced EAE, a model in which antigen-specific B cells contribute to disease pathogenesis, which involves the reduced infiltration of inflammatory cells in the central nervous system (CNS). CCR6 is upregulated in T_H_17 cells and innate lymphoid cells (ILC) that produce IL-17 and IL-22 which suggests that CCR6 inhibition could lead to the depression of T_H_17 type inflammatory reactions. Further, the antagonization of CCR6 with mAb should be an effective strategy for the treatment of T_H_17 or T helper lymphocyte 22 (T_H_22) mediated inflammatory autoimmune diseases, giving us the opportunity to selectively inhibit inflammatory cytokines, like interferon-gamma (IFN-γ) and interleukin-21 (IL-21), which are produced by CCR6^+^ T_H_17 cells under inflammatory conditions [20].

Posterior uveitis is an intraocular inflammatory disease that affects the uvea and the retina which can impair vision. Bromodomain extraterminal (BET) proteins have been recognized as potential inhibitors of EAE and now, of uveitis. In EAE, BET proteins act via the suppression of CD4^+^ T helper lymphocyte-1 (T_H_1) cells to reduce the disease severity. BET proteins are gene regulators that block the activity of the transcription factor T-bet, which, in turn, suppresses the proliferation of the T_H_1 subpopulation. A recent study on uveitis revealed that pharmacological blocking of T_H_17 cell differentiation occurs when BET proteins are used as inhibitors, which has been successful in attenuating inflammation in uveitis. Using both human and mouse in vitro cell cultures, they provided evidence that BET inhibitors suppress the expression of retinoic acid receptor related orphan nuclear receptor-gamma-t (RORγt) and significantly downregulate the T_H_17-associated genes interleukin 17A (IL-17A) and IL-22. The key finding was that BET inhibition markedly upregulated forkhead box P3 (FoxP3^+^) expression accompanied by lowered pathogenicity in vivo, suggesting that BET inhibition may switch retinal CD4^+^ T cell polarity from a T_H_17 to T_reg_ phenotype. Thus, it may represent a viable therapeutic entry point for inflammatory and autoimmune disorders which primarily depend upon the T_H_17/Treg axis for disease resolution [23].

Allergic conjunctivitis is an inflammatory disorder of the ocular surface that is characterized by Ig E and T helper lymphocyte-2 (T_H_2) driven allergic responses, which are marked by eosinophilic infiltration of the conjunctiva and involve the chemokine receptor CCR6. In mice induced with experimental allergic conjunctivitis (EAC), disease severity was compared between WT and CCR6 knockout (KO) models which showed the absence of CCR6-suppressed, allergen-specific immunoglobulin-E (Ig E) secretion, decreased mast cell and eosinophil accumulation, and therefore, minimized allergic conjunctival inflammation. Reduced inflammation was ascribed to reduced cytokine secretion from T_H_2 cell type in draining lymph nodes. Neutralization of the CCR6 ligand CCL20 with CCL20 neutralizing antibodies clearly repressed disease evaluating parameters, indicating that CCR6 might be important for the optimal development of T_H_2 responses as well as inflammation in EAC [24].

### 3.2. Integumentary System

Psoriasis is a chronic autoimmune skin disease that affects up to 3% of the world’s population. It is characterized by infiltration of inflammatory T cells to the skin in response to injury or autoantigens. Skin inflammation is caused by the cytokines of accumulated CCR6^+^ T cells and DCs which migrate towards the chemokine ligand CCL20 and other chemokines produced by keratinocytes and endothelial cells [19].

Animal models of psoriasis have already established the importance of the interleukin-23 (IL-23)/T_H_17 axis which is related to the CCR6–CCL20 receptor-ligand pair, thereby extending its importance to human disease. Human psoriatic skin invariably displays higher upregulation of CCR6/CCL20, in particular, with higher CCR6 expression on circulating peripheral blood mononuclear cells (PBMCs) than in normal skin. The treatment of psoriasiform skin inflammation induced by the drug Imiquimod (IMQ) with treated with an anti-hCCR6 mAb had a striking effect in preventing epidermal hyperplasia and dermal inflammation. This result is consistent with increased infiltration of IL-17 producing gamma delta T cells (γδ T cells) to psoriatic skin which was abrogated by anti-CCL20 mAb treatment. The inhibition of CCR6^+^ cell migration is considered more advantageous than anti-IL17/IL-17R therapies, because this would allow other cytokine pathways, such as IL-22, granulocyte macrophage colony stimulating factor (GM-CSF), and beta defensin (β-defensin) binding, to be targeted by the specific inhibition of CCR6^+^ cells [19,25].

CCR6 inhibition by a hitherto unidentified small molecule inhibitor named CCX9664 was published by a team of researchers experimenting with psoriasis. They reported that inhibition of the dominant T_H_17 receptor, CCR6, by CCX9664, an orally available inhibitor, was very effective in a preclinical in vivo model, leading to (i) reduced skin inflammation, which was correlated with (ii) a reduced number of IL-17 secreting T cells. Apparently, CCX9664 has proven its efficacy in models with equivalent results to those achieved with an antibody to the IL-17 receptor, which is described as a known human therapeutic target [26]. Another article by the same research team documented protection against psoriasiform-like disease in mice by a CCR6 antagonist through limited infiltration of CCR6^+^ leukocytes into the epidermis [27].

CCL20 neutralizing antibody was developed by Bouma and colleagues, and its efficacy in recruiting CCR6^+^ cells to sites of inflammation was tested using an experimental suction-blister model. In a randomized, placebo-controlled, first-in-human study, Bouma et al. employed GSK3050002, a humanized IgG1k antibody, which has been reported to be a potent CCR6 agonist, to assess target engagement and the ability to inhibit pro-inflammatory CCR6-expressing immune cell trafficking to the site of injury. The results demonstrated selective dose-dependent inhibition of CCR6^+^ T_H_17 cells by this novel antibody, supporting the further development of such CCL20 antagonists to treat autoimmune and inflammatory disorders [28].

### 3.3. Skeletal System

Data available from preclinical studies of research again highlights CCX9664 as a novel CCR6 antagonist that is capable of reducing disease severity in rheumatoid arthritis, a common autoimmune disease which affects the joints in the skeletal system. This chemokine receptor antagonist selectively blocks CCR6-mediated recruitment of T_H_17 cells to the sites of inflammation, providing an orally administrable inhibitor with minimal side effects [29]. Jaen et al., the same group of researchers, published another promising CCR6 antagonist, identified as CO339589, which inhibited CCL20-mediated chemotaxis and binding in a human natural killer (NK) cell line and in CCR6^+^ enriched human PBMC. It was identified as a highly potent molecule on both human and mouse CCR6 that is suitable for use in the treatment of diseases, rheumatoid arthritis, psoriasis, and multiple sclerosis (MS) [30].

### 3.4. Reproductive System

There is evidence that the CCR6–CCL20 axis may operate via the mitogen-activated protein kinase (MAPK), originally called extracellular signal-regulated kinases (ERK), and the stress-activated protein kinase C-JNK (jun kinase) pathways, which contribute to inflammatory responses in mammals. The MAPK/ERK pathway is a chain of proteins in the cell that communicates a signal from an extracellular receptor to the nuclear deoxyribonucleic acid (DNA) of the cell, initiating transcription. In endometriotic tissues and peripheral blood collected from patients with ovarian endometriomas, T_H_ 17 cells expressed CCR6, whereas CCL20 was secreted by the epithelial and stromal cells, thus promoting immune cell chemotaxis of the receptor-ligand pair. In this study, CCL20 caused selective migration of T_H_17 cells in the peripheral blood in a migration assay, while increased secretion of CCL20 was detected upon in vitro stimulation with interleukin-1 beta (IL-1β), TNF-α, and IL-17A in endometrial stromal cells. Interestingly, inhibition of the proteins p38 and p42/44 MAPKs and the stress-activated protein kinase c-Jun kinase suppressed the secretion of CCL20 that was increased by the pro-inflammatory cytokines IL-1β, TNF-α and IL-17A. This is suggestive of the fact that the CCR6/CCL20 system is involved in T_H_17 migration to endometriotic tissues and sets up inflammation that is upregulated by inflammation-inducing cytokines in the development of endometriosis. The protein inhibitors utilized in these experiments shed light on a possible therapeutic application that could become useful for curbing inflammatory disorders produced through CCCR6–CCL20 activation [31].

### 3.5. Gastrointestinal System

T_H_2 cytokine driven experimental food allergy was induced in a mouse model to evaluate both CCR6^+/+^ and CCR6^−/−^ cohorts, taking allergic diarrhea as a pre-clinical parameter. CCR6^−/−^ mice were protected from ovalbumin-induced diarrhea with significantly reduced T_H_2 cytokines, although allergen-specific Ig E production was not impaired, indicating that CCL20 regulates mucosal homeostasis and inflammatory trafficking of lymphocytes to the small intestine. The inhibition of CD4^+^T lymphocyte homing by treatment with FTY720, a sphingosine 1-phosphate 1 receptor agonist, shown to inhibit the egress of lymphocytes from mesenteric lymph nodes (MLN) and Peyer’s patches, did not impair allergic diarrhea in CCR6^+/+^ mice, suggesting the occurrence of reactivation of CCR6 expressing T cells locally within the small intestine. The study concluded a mast cell and Ig E independent role for CCR6^+^ T cells in the pathogenesis of allergic disease in the gastrointestinal tract [32].

Resveratrol (*trans*-3,4′,5-trihydroxystilbene) is a compound of the polyphenol group that is found abundantly in vegetables and various fruits, such as grapes, which showed discernible therapeutic potential in treating type I diabetes in a non-obese diabetic (NOD) mouse model. It is an activator of sirtuin 1, a nicotinamide adenine dinucleotide (NAD)–dependent sirtuin family deacetylase which is known to suppress T cell immune responses by deacetylating c-Jun, an activating protein-1 family transcription factor. A gene array analysis indicated a marked decrease in the expression of *Ccr6*, which encodes for CCR6, in mouse splenocytes treated with resveratrol that also displayed reduced CCR6 abundance. Further validation of the therapeutic efficacy of resveratrol in diabetes was shown by the accumulation of IL-17 –producing cells and CD11b^+^F4/80^high^ macrophages in the spleen and pancreatic lymph nodes but not in the pancreas itself, suggesting that resveratrol blocks CCR6 signaling and prevents migration of CCR6-bearing immune cells from peripheral lymphoid organs to the pancreas. Resveratrol is also known to prevent inflammation in mice via suppression of the nuclear factor kappa B (NF-kB) pathway to lower inflammatory cytokine release and induce apoptosis. Further, resveratrol produces inhibitory effects on MAPK, plasma creatine kinase, Src family tyrosine kinase, and phosphoinositide-3-kinase (PI3K) and has been used as medication in other autoimmune diseases, such as lupus, rheumatoid arthritis, and EAE [33].

## 4. Carcinoma Related Studies

A recently submitted application to patent an anti-cancer therapeutic intervention was based on cancers comprising cancer stem cells (CSCs) that express CCL20 and/or CCR6, including several cancers, such as pancreatic cancer, colorectal cancer, hepatocellular carcinoma, gastric cancer, lung cancer, head and neck cancer, and breast cancer. Inventors of this novel compound discovered the overexpression of CCL20 in CSCs and targeted their invention to inhibit CCL20 activity in cancers exhibiting cells with a CSC phenotype. Furthermore, the same group of researchers introduced a dual anti-cancer mechanism by modulating the CCR6/CCL20 axis in a tumor environment. They stated that the therapeutic neutralization of CCL20 (i) directly inhibits CSC activity and (ii) their tumorigenic ability, and (iii) relieves immunosuppression. They found that CCL20 secreted by tumor cells strongly influences the frequencies of immune-suppressive regulatory T_reg_ cells and T_H_17 populations, thereby supporting the fact that inhibition of the CCR6/CCL20 duo presents a novel therapeutic strategy in cancer treatment [17,34,35].

As is already evident, CCR6 and CCL20 have roles in the metastasis of advanced cutaneous T cell lymphoma (CTCL), demonstrating that CCR6 activation innervates the signal transducer and activator of transcription 3 (STAT3) pathway, mediating the transcription of CCL20, which leads to CTCL lymphomagenesis. Nutrient-dependent in vitro migration of CTCL cells was downregulated following transient knockdown of STAT3, CCL20, and CCR6 and administration of the anti CCL20 antibody. When examining the in vivo effect of neutralizing the CCL20 antibody in Xenografted SCID mice inoculated with CTCL cells, the group noted significantly prolonged survival where normally, they were expected to die with CTCL metastasis into multiple organs [36].

A second study on cutaneous T cell lymphoma documented inhibition of CCR6 by micro RNA-150 (MiR-150) leading to strong downregulation of tumor metastasis. MicroRNAs (miRNA) are a class of small regulatory RNA molecules which pair with target messenger RNAs to repress their productive translation and whose presence is significantly low in advanced CTCL cells. Altered expression of microRNA results in hematologic malignancies (lymphomas/leukemias) and tumorigenesis. MiR-150 was found to downregulate CCR6 which, in turn, reduced metastasis in an immune deficient mouse model, while CTCL cells displayed upregulation of IL-22 linked to increased stimulation of CCL20 and its binding to CCR6, thereby enhancing their multi-directional migration potential [37].

Chemokine/chemokine receptor mediated activation of ERK (extracellular signal-regulated kinase) signaling cascade (Akt, ERK1/2, stress-activated protein kinase/c Jun N terminal kinase MAPKs) has been identified to play crucial roles in cancer cell proliferation and invasiveness. In a clinical study of lung adenocarcinoma, colony formation capacity, ERK signaling and chemokine production were assessed and the colony forming capacity of cells taken from patients was shown to increase following CCL20 stimulation and depended, in part, on the ERK phosphorylation pathway. Carcinoma-associated lung fibroblasts communicate locally derived signals by interacting with chemokines and their receptors, resulting in carcinogenesis. Hence, the CCR6/CCL20 axis was found to be engaged in the proliferation and migration of cancer cells via autocrine or paracrine mechanisms, and it was suggested that disruption of the functions of this chemokine duo may offer a promising strategy to treat cancers in the lung [38].

The tumor microenvironment is precisely where the tumor cells begin to interact with the host immune system. It provides the location where the interactions between chemokine receptors and their ligands are modulated and which determines the type of immune cell subsets that are recruited towards the tumor. Thus, the chemokines distinctly influence tumor growth as well as its treatment options. This is mainly due to the degree of redundancy exhibited by the chemokine superfamily which allows the binding of multiple ligands to different receptors. When targeting a single chemokine receptor and ligand pair as a therapeutic measure in clinical trials, most of the time, remarkable progress is not produced because the chemokines bind to multiple receptors found on different types of cells. This brings about a diverse innervation of immune signaling networks, thereby controlling and sometimes compromising the immunotherapeutic outcome. Tumor immune responses are largely dependent upon the chemokines expressed by the tumor cells, other immune cell subsets, and non-immune cells, such as vascular endothelial cells. The tumor microenvironment is responsible for setting the pace for tumorigenesis, stem cell-like properties expressed by cancer cells, survival, invasiveness, and metastasis of cancer cells [39,40].

Tumor immunity is regulated by the trafficking of different lymphocyte subsets into the tumor microenvironment which helps to modulate several key chemokine signal transduction pathways. The activity of lymphocytes, particularly, in secreting effector cytokines synergistically with the chemokine activation has provided several immunotherapeutic avenues in the treatment of cancer. The cytotoxic CD8^+^ T cells are well known for their anti-tumor immunity, whereby they respond to tumor-associated antigens by releasing cytotoxic molecules, such as granzyme B and perforin. Similarly, interferon-gamma (IFN-γ) releasing T_H_1 cells and natural killer (NK) cells have demonstrated anti-tumor potency. The recruitment of T_H_17 cells expressing their signature chemokine, CCR6, is said to be polyfunctional within the tumor microenvironment, because these vells possess stem-like properties and mediate anti-tumor activity via the engagement of CD8^+^ T cells, NK cells, and DCs to sites of inflammation and cancer. In contrast to the above observations, T_H_22 cells that express CCR6 promote and support the progression of colon, pancreatic, and hepatocellular carcinomas, as also evidenced by pro-tumorigenic IL-22 in two murine colon tumor models. Hence, CCR6–CCL20 axis-mediated migration of T_H_22 into the tumor microenvironment is known to increase tumor proliferation. Similarly, regulatory T cells and B cells, which also express CCR6, have displayed known tumorigenic properties. DCs, macrophages, B cells, and T helper subsets are all recruited into the tumor microenvironment via the CCR6–CCL20 axis and other chemokine networks. They produce anti-tumor immunity by interacting with T cells and concomitantly produce tumor development by interacting with stem-like tumor cells and stromal cells. The other immunotherapies which may be effective against tumor progression include treatment with epigenetic modulators that upregulate effector T cell infiltration into the tumors, enhancement of the action of the programmed death-ligand 1 (PD-L1) checkpoint blockade, and adoptive transfer of T cells into tumorigenic mice [39,40].

## 5. Conclusions

This review summarized the roles of different CCR6–CCL20 inhibitors that have been investigated to date for their potency to modulate immune activation and therapeutic mechanisms in a number of inflammatory diseases and cancers. There is a dearth of deeper knowledge of the exact mechanisms of the CCR6–CCL20 axis. Extensive studies into immune and therapeutic functions of the CCR6–CCL20 axis with the use of novel inhibitors need to gain momentum, as there is a profound gap in immunological research into the treatment options for autoimmune and inflammatory disorders. New interventions with synthetic cellular, molecular, or nanoparticle inhibitors and derivatives of herbal and anti-toxic metabolites could be utilized to mimic or block the numerous biochemical pathways through which the CCR6–CCL20 axis is involved in disease promotion. Given the austere immune regulation inducted by CCR6 and CCL20 partners in skewing the T_H_17 and T_reg_ populations, tilting immune homeostasis into inflammation, even invention with a vaccine may become possible to boost T and B memory lymphocyte subsets of choice coupled with selective inhibition of these two chemokines. Comprehensive randomized, placebo-controlled, clinical trials on the therapeutic use of the CCR6–CCL20 axis could become useful to thoroughly evaluate the benefits and disadvantages of the inhibitors of this axis in humans.

## Figures and Tables

**Table 1:** Types of inhibitors used against the CCR6–CCL20 axis, their common names, the immune cells involved, experimental outcomes, and diseases involved.

Type of Inhibitor	Name of Inhibitor	Nature of Inhibitor	Immune Cells/Cytokines Involved	Experimental Outcome	Name of Disease/Model	Ref
Anti-hCCR6 mAbs		CCR6 Inhibitor	T_H_17, B cells	Reduced inflammatory cell infiltration	Experimental Autoimmune Encephalitis (EAE)	[20]
Anti-CCR6 Abs	CO339589	CCR6 inhibitor	NK cells PBMC	Reduced disease	RA, psoriasis, MS	[30]
Anti CCL20 Abs	WO2017011559A1	CCL20 inhibitor	T_H_17, T_reg_	Inhibits CSC activity, tumorigenesis	Carcinoma	[17]
	GSK3050002	CCL20 inhibitor	T_H_17	Reduced cell chemotaxis	Suction blister in skin	[28]
		CCL20 inhibitor	γδ T cells	Reduced immune cell infiltration to skin	Imiquimod (IMQ) induced psoriasiform dermatitis	[19]
Anti-TNF-α Abs	Infliximab Tocilizumab	Indirect CCL20 inhibitor	Synoviocytes	Inhibits TNF-α induced CCL20 upregulation	RA	[18]
Inhibitors of p38, 42, 44	MAPK Inhibitors	Indirect CCL20 inhibitor	T_H_17	Reduced pro-inflammatory cytokines, suppressed CCL20	Ovarian endometritis	[31]
Partial Agonists	AOP-RANTES	Partial CCR6 inhibitor	T_H_17	Reduced inflammatory cell chemotaxis	Psoriasiform dermatitis	[19]
Bispecific Abs	Humanised IgG-like BsAb	CCR6 and CXCR3 inhibitors	T_H_17	Blocks cell chemotaxis	Murine model	[20]
BET Proteins		Gene regulator	T_H_17, T_reg_	Reduced inflammation	Posterior uveitis	[23]
Small Molecule Inhibitors	CCX9664	CCR6 inhibitor	T_H_17	Reduced disease	Rheumatoid arthritiis (RA)	[29]
Botanical Inhibitors	EGCG, Gallotannin	CCL20 inhibitor	T_H_17	Inhibited cell chemotaxis	Rhesus Macaque model	[21]
Sphingosine 1 PO4 R agonist	FTY720		CD4^+^T Cells	Inhibited egress of lymphocytes from LNs	Allergic diarrhea	[32]
microRNA	MiR-150	CCR6 inhibitor	CTCL cells	Reduced metastasis	Cutaneous T cell lymphoma (CTCL)	[37]
Polyphenol compound	Resveratrol	Sirtuin-1 activator	T_H_17, macrophages	Reduced disease, inflammatory cytokines	Type-1 diabetes, RA, lupus, EAE	[33]
CCR6R Peptide Mimetics	ECL-2	CCL20 onhibitor	CCR6^+^ cells	Reduced cell chemotaxis	Rhesus Macaque model	[21]

Legend: MAPK—mitogen activated protein kinase, p—protein, CCL20—CC chemokine ligand 20, mAbs—monoclonal antibodies, BsAb—bispecific antibodies CCR6—CC chemokine receptor 6, hCCR6—humanised CCR6, Abs—antibodies, IL—interleukin, TNF-α—tumor necrosis factor-alpha, BET—bromodomain extraterminal proteins, PO_4_—phosphate, R—receptor, RNA—ribonucleic acid, AOP-RANTES—amino oxy pentane regulated on activation, normal T cell expressed and secreted. NK—natural killer, PBMC—peripheral blood mononuclear cells, IMQ—Imiquimod, T_H_17—T helper subset 17, T_reg_—regulatory T reg cell, γδ T cell—gamma delta T cell, EGCG—Epigallocatechin gallate, Ref—references to inhibitors, MS—multiple sclerosis, RA—rheumatoid arthritis.

## References

[1] Lee A.Y.S., Eri R., Lyons ABGrimm M.C., Korner H. (2013). CC chemokine ligand CCL20 and its cognate receptor CCR6 in mucosal T cell immunology and inflammatory bowel disease: Odd couple or axis of evil?. Front. Immunol..

[2] Griffith J.W., Sokol C.L., Luster A.D. (2014). Chemokines and chemokine receptors: Positioning cells for host defense and immunity. Annu. Rev. Immunol..

[3] Zlotnik A., Yoshie O., Nomiyama H. (2006). The chemokine and chemokine receptor super families and their molecular evolution. Genome Biol..

[4] Basheer W., Kunde D., Eri R. (2013). Role of chemokine ligand CCL20 and its receptor CCR6 in intestinal inflammation. Immunol. Infect. Dis..

[5] Ranasinghe R., Eri R. (2018). CCR6–CCL20-Mediated Immunologic Pathways in Inflammatory Bowel Disease. Gastrointest. Disord..

[6] Ranasinghe R., Eri R. (2018). CCR6–CCL20 Axis in IBD: What Have We Learnt in the Last 20 Years?. Gastrointest. Disord..

[7] Lu M.Y., Lu S.S., Chang S.L., Liao F. (2018). The phosphorylation of CCR6 on distinct Ser/Thr residues in the carboxyl terminus differentially regulates biological function. Front. Immunol..

[8] Scheerens H., Hessel E., deWaal-Malefyt R., Leach M.W., Rennick D. (2001). Characterization of chemokines and chemokine receptors in two murine models of inflammatory bowel disease: IL-10-/- mice and Rag-2-/- mice reconstituted with CD4+CD45RBhigh T cells. Eur. J. Immunol..

[9] Teramoto K., Miura S., Tsuzuki Y., Hokari R., Watanabe C., Inamura T., Ogawa T., Hosoe N., Nagata H., Ishii H. (2005). Increased lymphocyte trafficking to colonic microvessels is dependent on MAdCAM-1 and C-C chemokine mLARC/CCL20 in DSS –induced mice colitis. Clin. Exp. Immunol..

[10] Atreya R., Neurath M.F. (2010). Chemokines in inflammatory bowel diseases. Dig. Dis..

[11] Pezoldt J., Huehn J. (2016). Tissue specific induction of CCR6 and Nrp1 during early CD4+ T cell differentiation. Eur. J. Microbiol. Immunol. (Bp).

[12] Ranasinghe R., Eri R. (2018). Pleiotropic immune functions of chemokine receptor 6 in health and disease. Medicines.

[13] Lee AYPhan T.K., Hulett MDKörner H. (2015). The relationship between CCR6 and its binding partners: Does the CCR6-CCL20 axis have to be extended?. Cytokine.

[14] Proudfoot A.E. (2002). Chemokine receptors: Multifaceted therapeutic targets. Nat. Rev. Immunol..

[15] Ito T., Carson W.F., Cavassani K.A., Connett J.M., Kunkel S.L. (2011). CCR6 as a mediator of immunity in the lung and gut. Exp. Cell Res..

[16] Comerford I., Bunting M., Fenix K., Haylock-Jacobs S., Litchfield W., Harata-Lee Y., Turvey M., Brazzatti J., Gregor C., Nguyen P. (2010). An immune paradox: How can the same chemokine axis regulate both immune tolerance and activation? CCR6/CCL20, a chemokine axis balancing immunological tolerance and activation in autoimmune disease. Bioessays.

[17] Van Vlerken-Ysla L.E., Rios-Doria J., Moynihan J., Shan L., Hollingsworth R.E., Herbst R., Hurt E.M. (2017). Abstract 4779: Targeting the CCL20-CCR6 axis as a novel opportunity to simultaneously modulate cancer stem cells and the tumor–immune infiltrate by a dual anti-cancer mechanism. Cancer Res..

[18] Lee A.Y.S., Korner H. (2014). CCR6 and CCL20: Emerging players in the pathogenesis of rheumatoid arthritis. Immunol. Cell Biol..

[19] Getschman A.E., Imai Y., Larsen O., Peterson F.C., Wu X., Rosenkilde M.M., Hwang S.T., Volkman B.F. (2017). Protein engineering of the chemokine CCL20 prevents psoriasiform dermatitis in an IL-23 –dependent murine model. Proc. Natl. Acad. Sci. USA.

[20] Robert R., Juglair L., Lim E.X., Ang C., Wang C.J.H., Ebert G., Dolezal O., Mackay C.R. (2017). A fully humanized IgG-like bispecific antibody for effective dual targeting of CXCR3 and CCR6. PLoS ONE.

[21] Klamar C.R. (2011). Development of a Robust and Improved System for Studying Interactions between CCL20 and CCR6 Using Both Recombinant and Chemically Synthesized Rhesus Macaque Chemokines. Master’s Thesis.

[22] Liston A., Kohler R.E., Townley S., Comerford I., Caon A.C., Webster J., Harrison J.M., Swann J., Clark-Lewis I., Korner H. (2009). Inhibition of CCR6 function reduces severity of experimental autoimmune encephalomyelitis via effects on the priming phase of the immune response. J. Immunol..

[23] Eskandarpour M., Alexander R., Adamson P., Calder V.L. (2017). Pharmacological inhibition of bromodomain proteins suppresses retinal inflammatory disease and downregulates retinal Th 17 cells. J. Immunol..

[24] Chung S.H., Chang S.Y., Lee H.J., Choi S.H. (2015). The C-C chemokine receptor 6 (CCR6) is crucial for Th2 –driven allergic conjunctivitis. Clin. Immunol..

[25] Campbell J.J., Ebsworth K., Ertl L.S., McMahon J.P., Newland D., Wang Y., Liu S., Miao Z., Dang T., Zhang P. (2017). IL-17-secreting γδT cells are completely dependent upon CCR6 for homing to inflamed skin. J. Immunol..

[26] Ebsworth K., Ertl L.S., Wang H., Campbell J.J., McMahon J.P., Zhang P., Charo I.F., Schall T.J. 521 Chemokine receptor inhibition as a novel therapeutic approach for psoriasis. Proceedings of the Annual Meeting of Society for Investigative Dermatology.

[27] Campbell J.J., Ebsworth K., Ertl L., McMahon J.P., Zhang P., Singh R., Schall T.J. (2017). 669 chemokine receptor CCR6 antagonist reverses psoriaform dermatitis by preventing accumulation of γδT 17 cells in skin. J. Investig. Dermatol..

[28] Bouma G., Zamuner S., Hicks K., Want A., Oliveira J., Choudhury A., Brett S., Robertson D., Felton L., Norris V. (2017). CCL20 neutralization by a monoclonal antibody in healthy subjects selectively inhibits recruitment of CCR6+ cells in an experimental suction blister. Br. J. Clin. Pharmacol..

[29] Dairaghi D., Zhang P., Leleti M., Berahovich R., Ebsworth K., Ertl L., Miao S., Miao Z., Seitz L., Tan J. et al. Inhibition of chemokine receptors CCR1 and CCR6 as promising therapies for autoimmune diseases such as rheumatoid arthritis and psoriasis. In Proceedings of the ACR/ARHP Annual Meeting.

[30] Jaen J.C., Dairaghi D., Leleti M., Powers M.J.P., Wang Y., Zhang P., Schall T.J. (2013). FRI0002 Inhibition of chemokine receptors CCR1 and CCR6 as promising therapies for autoimmune diseases such as rheumatoid arthritis. Ann. Rheumetic Dis..

[31] Hirata T., Osuga Y., Takamura M., Kodama A., Hirota Y., Koga K., Yoshino O., Harada M., Takemura Y., Yano T. (2010). Recruitment of CCR6—Expressing Th17 cells by CCL20 secreted from IL-1 beta, TNF- alpha, and IL-17A-stimulated endometriotic stromal cells. Endocrinology.

[32] Blazquez A.B., Knight A.K., Getachew H., Bromberg J.S., Lira S.A., Mayer L., Berin M.C. (2010). A functional role for CCR6 on pro-allergic T cells in the gastrointestinal tract. Gastroenterology.

[33] Lee S.M., Yang H., Tartar D.M., Gao B., Luo X., Ye S.Q., Zaghouani H., Fang D. (2011). Prevention and treatment of diabetes with resveratrol in a non-obese mouse model of type 1 diabetes. Diabetologia.

[34] Liu J., Ke F., Xu Z., Liu Z., Zhang L., Yan S., Wang Z., Wang H., Wang H. (2014). CCR6 is a prognostic marker for overall survival in patients with colorectal cancer and its overexpression enhances metastasis in vivo. PLoS ONE.

[35] Marsigliante S., Vetrugno C., Muscella A. (2013). CCL20 induces migration and proliferation on breast epithelial cells. J. Cell Physiol..

[36] Ikeda S., Kitadate A., Ito M., Abe F., Nara M., Watanabe A., Takahashi N., Miyagaki T., Sugaya M., Tagawa H. (2016). Disruption of CCL20-CCR6 interaction inhibits metastasis of advanced cutaneous T cell lymphoma. Oncotarget.

[37] Ito M., Teshima K., Ikeda S., Kitadate A., Watanabe A., Nara M., Yamashita J., Ohshima K., Sawada K., Tagawa H. (2014). MicroRNA-150 inhibits tumor invasion and metastasis by targeting the chemokine receptor CCR6, in advanced cutaneous T-cell lymphoma. Blood.

[38] Zhang X.P., Hu Z.J., Meng A.H., Duan G.C., Zhao Q.T., Yang J. (2017). Role of CCL20/CCR6 and the ERK signaling pathway in lung adenocarcinoma. Oncol. Lett..

[39] Nagarsheth N., Wicha M.S., Zou W. (2017). Chemokines in the cancer microenvironment and their relevance in cancer immunotherapy. Nat. Rev. Immunol..

[40] Peng D., Kryczek I., Nagarsheth N., Zhao L., Wei S., Wang W., Sun Y., Zhao E., Vatan L., Szeliga W. (2015). Epigenetic silencing of TH1 -type chemokines shapes tumor immunity and immunotherapy. Nature.

